# A framework for designing AI systems that support community wellbeing

**DOI:** 10.3389/fpsyg.2022.1011883

**Published:** 2023-01-04

**Authors:** Willem van der Maden, Derek Lomas, Paul Hekkert

**Affiliations:** Department of Human Centered Design, Faculty of Industrial Design Engineering, Delft University of Technology, Delft, Netherlands

**Keywords:** human-centered design, community wellbeing, artificial intelligence, cybernetics, wellbeing economy, feedback loop, human values

## Abstract

**Introduction:**

Designing artificial intelligence (AI) to support health and wellbeing is an important and broad challenge for technologists, designers, and policymakers. Drawing upon theories of AI and cybernetics, this article offers a design framework for designing intelligent systems to optimize human wellbeing. We focus on the production of wellbeing information feedback loops in complex community settings, and discuss the case study of My Wellness Check, an intelligent system designed to support the mental health and wellbeing needs of university students and staff during the COVID-19 pandemic.

**Methods:**

The basis for our discussion is the community-led design of My Wellness Check, an intelligent system that supported the mental health and wellbeing needs of university students and staff during the COVID-19 pandemic. Our system was designed to create an intelligent feedback loop to assess community wellbeing needs and to inform community action. This article provides an overview of our longitudinal assessment of students and staff wellbeing (n = 20,311) across two years of the COVID-19 pandemic.

**Results:**

We further share the results of a controlled experiment (n = 1,719) demonstrating the enhanced sensitivity and user experience of our context-sensitive wellbeing assessment.

**Discussion:**

Our approach to designing “AI for community wellbeing,” may generalize to the systematic improvement of human wellbeing in other human-computer systems for large-scale governance (e.g., schools, businesses, NGOs, platforms). The two main contributions are: 1) showcasing a simple way to draw from AI theory to produce more intelligent human systems, and 2) introducing a human-centered, community-led approach that may be beneficial to the field of AI.

## Introduction

As the use of Artificial Intelligence (AI) continues to advance, researchers are increasingly exploring its potential to support and enhance mental health and wellbeing. With its ability to analyze large amounts of data and make complex decisions, AI has the potential to provide valuable insights and support to individuals and communities seeking to improve their mental health and wellbeing (D’Alfonso, 2020). In this paper, rather than viewing “AI for wellbeing” as a specialized interest in the mental health community, we argue that all ethical AI systems should have the implicit objective of enhancing human wellbeing. This is in line with the Institute of Electrical and Electronics Engineers standards review on ethical design: “by aligning the creation of [AI] with the values of its users and society we can prioritize the increase of human wellbeing as our metric for progress in the algorithmic age” (Shahriari and Shahriari, 2017).

While ‘AI for Wellbeing’ often refers to the use of AI tools, such as chatbots, to support mental health and wellbeing, this article focuses on using theories developed by AI researchers to better understand how large-scale systems can be designed to enhance and support wellbeing outcomes.

For example, the European Commission defines AI Systems as “systems that display intelligent behavior by analyzing their environment and taking actions—with some degree of autonomy—to achieve specific goals” (2019, p. 1).

More specifically, a popular AI textbook defines the field of artificial intelligence as the “study and design of intelligent agents” where an agent is anything that can be viewed as perceiving its environment through sensors and acting upon that environment through actuators (Russell and Norvig, 2022). For such an agent to be considered intelligent, it must possess the “ability to select an action that is expected to maximize [a] performance measure…an agent that is assigned an explicit “goal function” is considered more intelligent if it consistently takes actions that successfully maximize its programmed goal function” (Russell and Norvig, 2022, p. 58). Based on these criteria, AI systems must possess the ability to sense their environment, act on their environment, measure an explicit goal state in the environment (i.e., a performance measure or objective function), and use sense data to choose actions likely to improve that performance measure—see Box 1 for a proposed framework based on these theoretical propositions. In brief, this theory suggests that it may be inevitable that future wellbeing-aligned AI systems will necessarily need mechanisms for assessing human wellbeing.

**BOX 1** A framework for designing AI Systems, based on a definition of AI focused on the “ability to select an action that is expected to maximize [a] performance measure” (Russell and Norvig, 2022).

**Table:** 

	**Step**	**Description**
1	Task Environment	Define the task environment in which the AI system will be used and the requirements for success in that environment
2	Performance Measures	Develop performance measures or objective functions that quantify the goals of the system.
3	Action space	Identify the set of possible actions that the AI system can take in the environment and the set of possible states that can result from those actions
4	Sensors	Define a set of features that can be used to describe the state of the sensed environment
5	Algorithms	Define a set of algorithms that can be used to map the features of the environment to the actions that the AI system can take, for the purpose of optimizing the objective function—where the algorithm need not be software (Gillespie, 2014)
6	Implementation	Implement the system within the constraints of the environment, the users, and other stakeholders—the designers should remain an integral part of the implementation procedure and monitor performance (Norman and Stappers, 2015)
7	Refine	Based on feedback from users and other stakeholders, refine the system as necessary to improve performance

This paper provides a demonstration of developing context-sensitive wellbeing assessments that may inform the design of future AI system assessments of wellbeing through community-led design. I.e., we present the case study of My Wellness Check: an intelligent system that measures human wellbeing in order to optimize and support the needs of university students and staff during the COVID-19 pandemic. Designed in collaboration with students, staff, and mental health professionals, the My Wellness Check system provided a governing feedback loop capable of assessing community wellbeing needs and informing community action. Based on theoretically-derived factors of wellbeing as well as factors defined by community participants, My Wellness Check produced real-time insights into community wellbeing that were used to inform actions at various levels of the university, from top administrators to individual students. We share data from a longitudinal deployment of My Wellness Check to nearly 30,000 students and staff across 2 years of the COVID-19 pandemic. To evaluate our system, we share the results of a controlled experiment comparing our community-led wellbeing assessment to other wellbeing assessments. This shows that our community-led designs generated greater predictive value and a significantly better user experience. While our results cannot serve as proof of efficacy for the performance of our entire system, it does show the benefits of our community-led design process.

Schools, businesses, NGOs, social platforms and other large-scale governing systems may wish to systematically improve the wellbeing of the people they serve. Our work aims to provide insights that can generalize to these different contexts. Rather than designing a fully autonomous system (for example, a chatbot to help provide students with mental health recommendations), we focused on introducing an intelligent feedback loop to an existing sociotechnical system.

### Paper overview

In the first part of this article, we provide an overview of the concept of “designing AI for Wellbeing” and review some related efforts. We then discuss several methods and ideas popular within the field of human-centered design, such as participatory design, community-led design, systems thinking, and cybernetic thinking to address some of the challenges of designing AI for Wellbeing.

In the second part of the paper, we describe the specific context and the design of My Wellness Check. We then present data from multiple assessments of wellbeing over the period of the pandemic. Following a description of the design of the system, we present the design and implementation of a controlled experiment to evaluate our context-sensitive assessment. Following the presentation of the results of this experiment, we then reflect upon our design framework and suggest opportunities for future research in the design of AI systems for Community Wellbeing.

## Related work: Designing AI for wellbeing

There is a small, but growing, body of work on the use of AI for wellbeing or mental health, much of which focuses on the use of AI for health monitoring and personalized health advice. Often these services are delivered through the use of virtual agents, chatbot, wearables and other IoT technologies (see review by Shah and Chircu, 2018). According to D’Alfonso (2020) the three main applications of AI in mental health are: (1) personal sensing or digital phenotyping; (2) natural language processing of clinical texts and social media content; and (3) chatbots, while another review found opportunities for AI in mental health mainly related to self-tracking and AI assisted data analysis (Graham et al., 2019).

A 2020 Designing Interactive Systems (DIS) workshop on wellbeing offered the following summary of the field: “Most human-computer interaction (HCI) work on the exploration and support of mental wellbeing involves mobiles, sensors, and various on-line systems which focus on tracking users” (Sas et al., 2020). This reflects a focus on user-centered solutions for wellbeing, where wellbeing is conceptualized as the concern of an individual person.

In this paper, we present an alternative design objective: to support the wellbeing of a community of people. The wellbeing of a community can be understood as a multidimensional set of values, including economic, social, and environmental values, that impact people in a community (see review by Shah and Chircu, 2018). One advantage of this approach is that it does not require tracking individuals over time, which poses more risks from a data privacy and security perspective. Individual tracking, when it reveals deficits in wellbeing, may be damaging to individual self-image and produce feelings of guilt or disappointment (Chan et al., 2018).

A 2019 review of HCI technologies for wellbeing proposes the following: “We argue for an ethical responsibility for researchers to design more innovative mental health technologies that leverage less the tracked data and more its understanding, reflection, and actionability for positive behavior change.” (Sanches et al., 2019). By focusing on wellbeing at a community level (namely, the students and staff at a campus university), we can avoid data tracking issues and include diverse stakeholders that can assist with understanding the wellbeing data, reflecting upon it and formulating approaches for positive action.

As part of our community-focus, our work centers around two components of the system: assessments of community wellbeing needs and the design of interventions that target those needs. We are inspired by cybernetic theory to design our system to produce a wellbeing feedback loop that supports both top-down and bottom-up processes.This approach lends itself to our participatory and community-led design methods It also stands in contrast to the objective of developing an autonomous system that uses a black-box, algorithmic approach to intervene in the community. Finally, we use an iterative, longitudinal design approach that emphasizes improvements in the assessment of wellbeing and the processes taken to transform those assessments into action.

### Cybernetics: A conceptual framework for designing intelligent feedback loops

**Figure 1:**
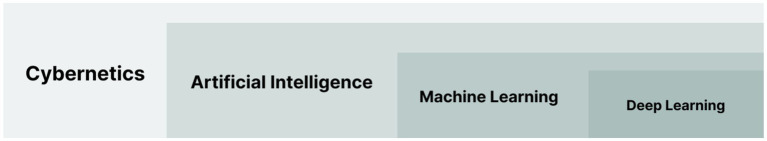
Representation of cybernetics as the conceptual predecessor of AI used with permission from Lomas J. D. et al. (2021).

Cybernetics has seen a resurgence of interest due to the increasing popularity of artificial intelligence and machine learning (Pangaro, 2017, 2021), and can be seen as its conceptual predecessor (Figure 1). Partially explaining this interest is the common (mis)conception that the purpose of artificial intelligence is to replace human intelligence with computational intelligence—also called “AI thinking” (van der Maden et al., 2022). As artificial intelligence does typically focus primarily on computational systems, cybernetics offers a conceptual framework for understanding the design of systems that are capable of purposeful (intelligent) behavior—regardless of whether the systems involved are natural, artificial, or a mix of the two.

Cybernetics can be described as the interdisciplinary study of the design of governing systems, both human and machine, that use sensors and actuators to achieve a goal. The word cybernetics comes from the Greek word κῠβερνήτης (kubernḗtēs), which means “steersman” or “governor” (note that the verb “to govern” also comes from this Greek root). Cybernetics has been used to help design everything from robots to organizations. It has also been used to study human cognition and social interactions.

**Box 2: Wellbeing theory.**

**Conceptualizing Wellbeing**

According to the World Health Organization, wellbeing is “a state of complete physical, mental, and social well-being, and not merely the absence of disease or infirmity” (World Health Organization, 1946). In other words, wellbeing is more than just being physically healthy—it also includes being mentally and emotionally healthy and feeling like you belong to and are supported by a community.

The academic literature consists of many ways to conceptualize and operationalize wellbeing. Some common dimensions of wellbeing include physical health, mental health, emotional health, social health, and spiritual health. While there is agreement among scholars, a strong consensus on the definition of the concept of wellbeing seems absent (Dodge et al., 2012). Academics criticize the field on the basis that definitions are heavily dependent on the cultural background of the researcher and the application area of the research (Alexandrova, 2012). Considering the conceptual dissensus, a review by Cooke et al. (2016) identifies four main areas of wellbeing literature which will be used as a framework in this paper.

*Hedonic* models of wellbeing focus on both pleasure and happiness. This field is pioneered by Ed Diener’s tripartite model of subjective wellbeing (Diener et al., 1985, 1999), which considers satisfaction with life, the absence of negative emotions, and the presence of positive emotions, as vital components of wellbeing. This perspective typically considers all three of these constructs when assessing wellbeing, although many researchers focus primarily on Diener’s “Life Satisfaction Score” (Cooke et al., 2016; Linton et al., 2016). *Eudaimonic* models of wellbeing offer research that tries to account for more than the pleasure of a satisfied life. For example, Ryff’s six-factor model of psychological wellbeing focuses on self-acceptance, positive relations with others, autonomy, environmental mastery, purpose in life, and personal growth (Ryff and Keyes, 1995; Ryff and Singer, 2006). Further, Martin Seligman’s wellbeing theory encompasses both perspectives (hedonic and eudaimonic) stating that wellbeing (or *flourishing*) can be conceptualized in terms of positive emotion, engagement, meaning, positive relationships, and accomplishment (PERMA) (Seligman, 2011). A third category of wellbeing research focuses on *quality of life* (QoL). Cooke et al. (2016) note that while this term is often used interchangeably with wellbeing, it should be seen as a separate category because research on QoL generally conceptualizes wellbeing to encompass models of physical, psychological, and social functions. It is often associated with wellbeing towards or during end of life and living with a disability. A common assessment instrument is the Quality of Life Inventory developed by Frisch et al. (1992). Lastly, Cooke et al. (2016) describe a fourth category that is called *wellness*. They note that wellness approaches are often rooted in counseling and tend to be broader and less clearly defined and not necessarily associated with assessment instruments. Rather, wellness practitioners focus on a holistic lifestyle that can include many areas of health and functioning including spiritual health.

**Community Wellbeing**Wellbeing is often understood as centered around individual experiences. However, wellbeing for a person is also dependent on a “set of interlocking issues and constraints and embedded in a dynamic social context.” (Phillips and Wong, 2017). In this vein, Musikanski and colleagues consider community wellbeing to be defined as encompassing the domains of community, culture, economy-standard of living (which includes housing, food, transportation and information and communication technology), education, environment, government, health, psychological well-being, subjective well-being and affect, time balance and work (2020, p.41). In line with this categorization, community wellbeing has been operationalized to encompass similar categories (vander Weele, 2019; vander Weele et al., 2020).

**Figure 2:**
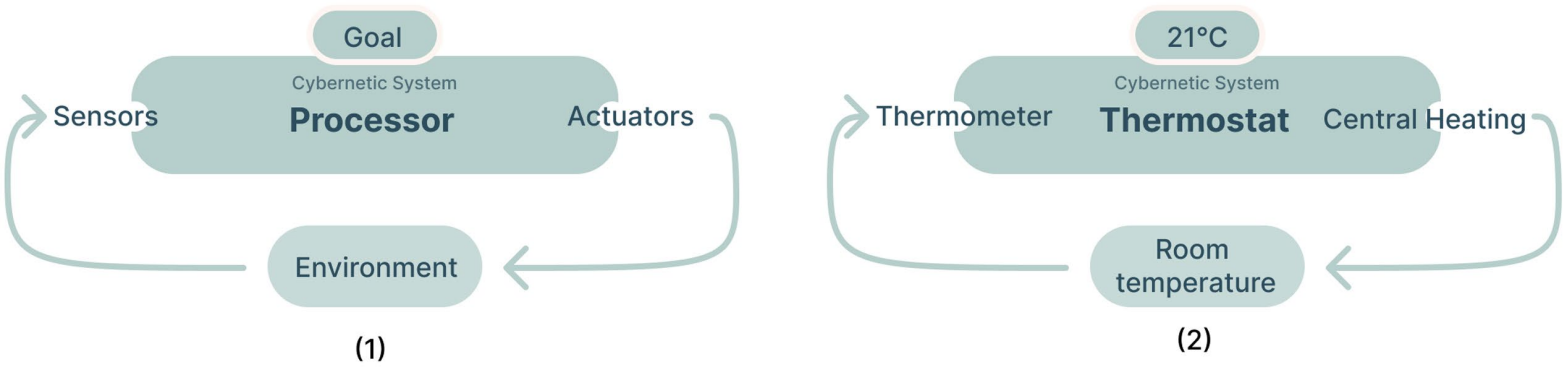
The schematic on the left (1) is an abstraction of a typical cybernetic system (adapted from Dubberly and Pangaro, 2007). The schematic on the right (2) shows a typical example of a cybernetic system, a thermostat.

A cybernetic system is a system where feedback loops are used to control the behavior of the system. At its simplest, a cybernetic system consists of just three parts: a controller, a sensor, and an effector or actuator. In a simple home thermostat, for example, the sensor is the temperature sensor, the effector is a switch that turns on a heater, and the controller is a mechanism that compares the sensor to a point set by the user. If the sensor value is below the set point, the controller turns on the heater, see Figure 2. If the sensor value is above the set point, the controller turns off the heater. More complex “smart thermostats” may have additional sensors (e.g., humidity, occupancy, etc.), and effectors (e.g., air conditioner, fan, etc.), and the controller may use a more sophisticated algorithm to determine when to turn devices on and off.

Cybernetic systems are not restricted to simple devices like thermostats, however. Cybernetic systems can be found in living organisms (e.g., the feedback loops that control blood sugar levels), in social systems (e.g., the feedback loops that govern the interactions between people), and in artificial intelligence systems (e.g., the feedback loops that allow a robot to learn from its mistakes).

**Figure 3:**
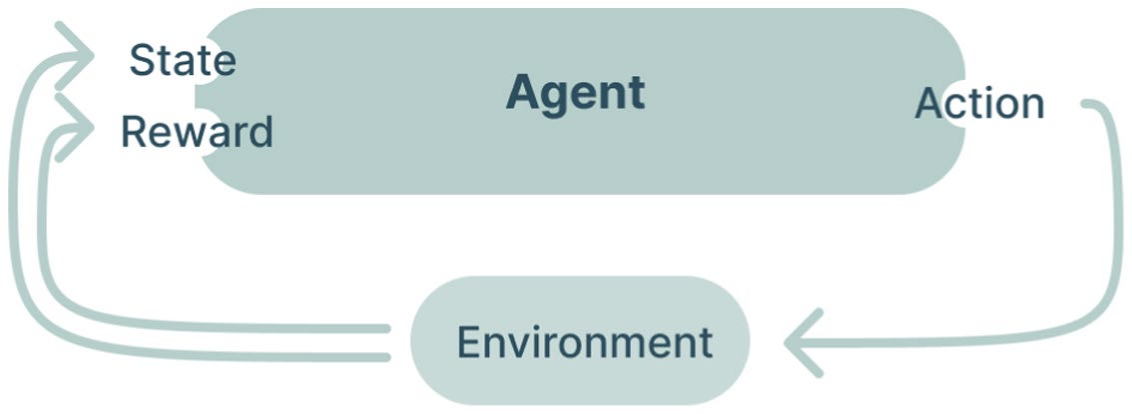
A schematic of a typical reinforcement learning algorithm (adapted from Sutton and Barto, 2018).

Cybernetics is closely related to the field of artificial intelligence. Both fields are concerned with the design of adaptive systems. A typical example is reinforcement learning, which is a machine learning method that uses rewards or punishment to train an agent to perceive and interpret its environment and take actions, see Figure 3. The fields also differ in their typical focus. For instance, cybernetics tends to be more concerned with understanding or designing feedback mechanisms that allow a system to govern its behavior, whereas artificial intelligence is more concerned with the design of algorithms that allow a system to learn from or adapt to its environment. Second, cybernetics tends to be more concerned with the design of natural systems (i.e., designing human governing systems), while artificial intelligence is more concerned with the design of computational systems.

Practically speaking, cybernetics offers a viewpoint for designing intelligent systems for governance that include both computers and people (e.g., Krippendorff, 2007, 2021; Glanville, 2009; Sweeting, 2016; Dubberly and Pangaro, 2019). Replacing human intelligence with computational automation is often not desirable, largely due to the special capacities of human interactions. Instead, there is a need to design systems, both natural and computational, that work together to create more intelligent behavior (i.e., more able to achieve goals in an uncertain environment). Cybernetics provides a means for conceptually uniting humans and artificial systems While keeping “humans in the loop” is a key design objective for many AI researchers, it is common for people to view artificial intelligence as an autonomous system that does not rely on human participation. It is as though, if human intelligence is still participating in the system, then the AI is not finished. Cybernetics may therefore offer a viewpoint for designing artificial intelligence in complex human systems where there is no desire to replace human intelligence with computational automation. This seemed especially apt in the context of supporting university administrators in supporting the wellbeing needs of their community during the COVID-19 pandemic.

It can be challenging to conceptualize the design of an AI system that makes such extensive use of human information processing and action. To conceptualize how an AI system can be designed in the context of a larger human system, we look to systems-thinking (e.g., Arnold and Wade, 2015) and cybernetic approaches (e.g., Wiener, 1961; von Foerster, 2003; Krippendorff, 2007). These perspectives point to how artificial systems may be designed to leverage human systems that are already functioning in a community, rather than trying to do everything autonomously. The cybernetic approach helps simplify the algorithmic design problem by focusing on a core process: generating a feedback loop between assessments of wellbeing and actions taken to enhance wellbeing. Furthermore, the cybernetics approach frees us from having to automate all processes into computational processes; we can design intelligence into a complex-sociotechnical system without having to make an entirely autonomous AI agent. Furthermore, we will show that such a system can be implemented rapidly and, over time, can be improved through iterative design, community feedback, and appropriate automation.

**Figure 4:**
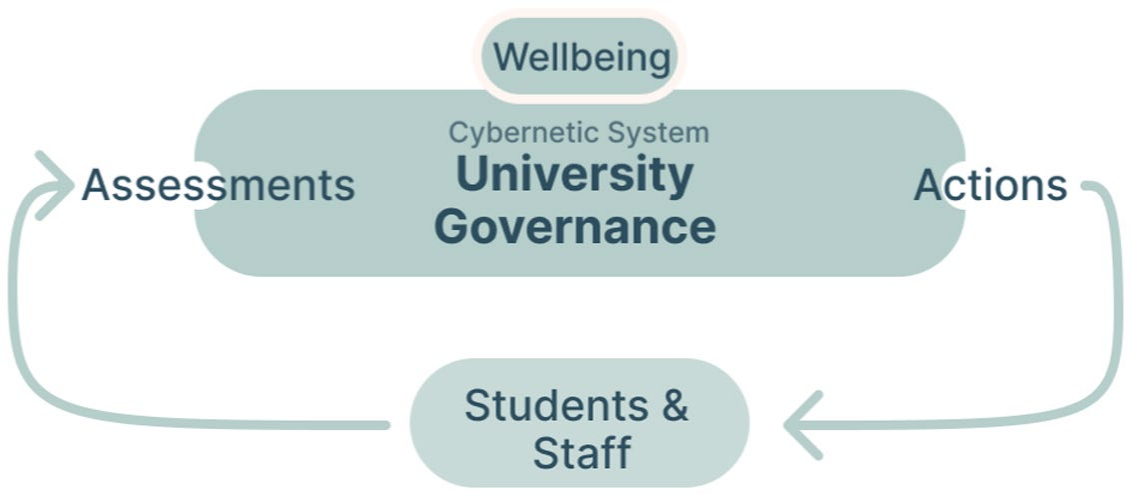
A schematic that visualizes the different components that were involved in the feedback loops in our context.

To summarize, based on theories of artificial intelligence and cybernetics, we sought to create an intelligent feedback loop capable of promoting community wellbeing. Figure 4 below visualizes the components that were involved in the feedback loops in our context. The design of the ability to *sense the state of the system* will be discussed next after which the process of defining the *action space* and *processor* (which we have explained are necessary for a cybernetic feedback loop) will be described.

## Case study: My wellness check, wellbeing at scale during COVID-19

The COVID-19 pandemic has been a major shock to societies around the world, with far-reaching consequences for mental health and wellbeing. In particular, the COVID-19 pandemic appears to have measurably reduced rates of human wellbeing around the world (Brodeur et al., 2021). The pandemic has also sparked interest in the role of large organizations in supporting wellbeing as an explicit criterion of organizational success. Universities, for instance, aim for a variety of organizational metrics of success (e.g., high graduation rates, large numbers of applicants, sustainability metrics, etc.). Increasingly, universities are recognizing student and staff wellbeing as explicit institutional priorities (Burns et al., 2020; Lomas D. et al., 2021).

During the onset of the COVID-19 pandemic in early 2020, the university administration asked us how they could best support the wellbeing of their staff and students who were now locked inside their homes due to government restrictions. This led to a two-year, iterative, community-led design process of a system that helped support mental-health and wellbeing. In this section we will address parts of that process that may translate to other contexts. The generalizability lies in the parts that have emerged from our cybernetic process—such as the context-sensitive assessment—and translate conceptually, not factually.

The most essential requirement of our system was to create *the ability to sense wellbeing needs in the community*. This “sensor” would be at the heart of any future AI system or cybernetic system for community wellbeing. Therefore, our primary goal was to design a wellbeing assessment instrument that was sensitive to the needs of our specific context—and capable of informing and motivating appropriate actions in response.

### Theoretical approach to wellbeing

To develop our sensor, we did not choose one particular theory of human wellbeing (discussed in Box 2), but rather took a syncretic approach and drew from multiple theoretical traditions (e.g., Diener et al., 1999; Ryff and Singer, 2006; Seligman, 2011). This was justified because the goal of our assessment differed from the typical goal of conventional psychological approaches to measuring human wellbeing (discussed in Box 3), which is to create an accurate and theoretically valid measurement instrument. Instead, our goal was to create an actionable assessment: an assessment purposefully created to help inform and motivate concrete actions in the community to promote wellbeing.

**Box 3: Wellbeing assessment.**

**Measuring Wellbeing**

In a recent review of 99 self-report assessments of wellbeing in adults (Linton et al., 2016), the authors note that there are a vast range of instruments based on different fundamental theories. In their review, they suggest that two of the most influential theories are *subjective wellbeing* from Diener et al. (1985, 1999) and *psychological wellbeing* from Ryff and Keyes (1995). They conclude that different instruments may be suitable depending upon the needs of the context. This sentiment is echoed in another recent review of 42 instruments (Cooke et al., 2016).

Despite the lack of convergence in academia, a recent McKinsey report on wellbeing in Europe states that “a consensus is nevertheless emerging on how best to measure well-being. Researchers now tend to ask a basic question: “Overall, how satisfied are you with your life nowadays?” (Allas et al., 2020). This question, based on Diener’s life satisfaction measure, is appealing because it is short and because it allows for comparison between populations and over time. However, this measure does not specifically provide information about what is wrong or what might help.

**Domain-specific Wellbeing**

While life satisfaction scores provide an excellent means for comparison, an assessment of wellbeing may be intended to *inform* useful actions to support improved wellbeing. For instance, measures of employee satisfaction are typically undertaken with the goal of improving employee satisfaction. Because of this, wellbeing assessments should ideally be sensitive to the needs of a particular domain. In a primary school setting, for instance, bullying may have a significant effect on a student’s wellbeing; in a company setting, work-life balance may have a significant effect on employee wellbeing. In both cases, a domain-specific measure can be more useful for informing actions that may help improve wellbeing in the specific context.

When efforts are made to assess wellbeing in specific domains, like work or school, we refer to this as a domain-specific wellbeing assessment (e.g., Renshaw et al., 2014; Renshaw and Bolognino, 2014; Gregory et al., 2019). For instance, the College Student Subjective Wellbeing Scale (CSSWQ) has been designed to assess a combination of relevant components for college students which the researchers refer to as “covitality” (Renshaw et al., 2014). The different components include Satisfaction with Academics, Academic Grit, School Connectedness, Academic Self-Efficacy and College Gratitude.

**COVID-19 and student wellbeing**

The COVID-19 pandemic and lockdowns had a significant impact on subjective wellbeing around the world (e.g., Aucejo et al., 2020; White and Van Der Boor, 2020; De Pue et al., 2021; Hu et al., 2021; Khan et al., 2021). Common topics studied include anxiety, loneliness, psychological stress, and post-traumatic stress disorder (Xiong et al., 2020). These effects may be especially amplified in university students as many tend to live in small housing, away from their families, and experience financial instability. Aside from that, students were expected to complete their educational goals as if it were a normal situation despite the many factors restricting them (e.g., internet connection, lack of jobs, family challenges) (Crawford, 2020). According to the literature, students suffered from decreased motivation (Tan, 2020), hopelessness (Pretorius, 2021), and depression (Fawaz and Samaha, 2021). In response, some publications suggest that mindsets should be changed: for instance, grit and gratitude (Bono et al., 2020) or optimism (Genç and Arslan, 2021) are offered as approaches to improve wellbeing and cope with the pandemic. These recommendations, however, do not directly indicate how communities or organizations might respond to improving student wellbeing.

**Box 4: Context-sensitivity, actionability, and assessment experience.**

**Context-sensitivity**

Socially disruptive events, such as a pandemic, can trigger changes in human values and their prioritization in society (Daher et al., 2017; Klenk and Duijf, 2021). Due to isolation and lockdowns during COVID-19, there seemed to be many factors that were previously not considered as critical to wellbeing. For instance, the experience of one’s home working-environment—factors such as ‘Wi-Fi quality’ or ‘a dedicated work desk’ are generally not considered by wellbeing assessment instruments. Yet, in the context of COVID-19, these factors became relevant to the wellbeing experience of community members. It is a general challenge for design research to identify the various mechanics that affect wellbeing (Fokkinga et al., 2020). Therefore, we needed a method that could identify important new factors—to identify if we were asking the right questions to the right people. This method should help identify what factors are *currently* actively impacting wellbeing in a manner that can point to where interventions should and can be designed.

**Actionability**

“Off-the-shelf” measures of wellbeing, mainly found in psychological literature (as discussed in the previous section), are oftentimes constructed primarily for validity and reliability—not *actionability*. What we define as actionability is the usefulness of a measure for informing helpful actions. For example, the Satisfaction with Life Scale (SWLS; Pavot and Diener, 2009) has been proven to be a strong cross-cultural measure of a person’s wellbeing, but it is not designed to indicate how to improve wellbeing within a specific context. To illustrate, imagine you are an administrator aiming to improve the wellbeing of your members in your organization, knowing that the average member in your community has an SWLS score of 21, and a PANAS score of 26, does not immediately inform you on where you might take actions to improve these scores. The scores must be related to contextual factors in order to be meaningful. On the other hand, in the domain of universities, the College Student Subjective Wellbeing Questionnaire (CSSWQ; Renshaw and Bolognino, 2014) may be more actionable than a general measure (such as the SWLS) due to the granularity of its questions. But, despite this granularity, the questions do still not directly point to opportunities for taking action. Current measures of wellbeing may be reliable and valid and yet the information provided by these measures may not be sufficiently concrete that communities might use to take action to support improved wellbeing. Note that it is not specifically items that provide more *actionable* information, it is the assessment instrument as a whole, combining “off-the-shelf” measures with contextualized items. Hence the term context-sensitive assessment—not measure—of wellbeing.

**Assessment Experience**

The assessment experience is important for two basic reasons. First, a positive experience can lead to improved participant engagement and data quality (Stocké and Langfeldt, 2004; Baumgartner et al., 2021). Second, the experience of assessing wellbeing has the potential to offer an intervention in and of itself. Namely, reviewing different facets of one’s own life has the potential to lead to constructive change and experiences of improved wellbeing. While this second rationale for improving the wellbeing assessment experience was not quantitatively evaluated in this study, it was a driving motivation for the design of My Wellness Check.

Popular measures of wellbeing often focus on generalization. That is, they seek to validate a measure that can be used for comparing multiple contexts. Diener’s single item life satisfaction measure is a good example: on a scale of 0–10, how satisfied are you with your life as a whole? This measure (and its many variants) has been extremely useful for comparing wellbeing in different contexts. This measure is “actionable” insofar as a low score shows that something should be done. However, attributing the low score to specific causes is problematic—making it difficult to take specific actions. For this reason, we sought to devise new measures of wellbeing that were highly specific to the context of the community we sought to serve. We anticipated that a context-sensitive assessment would be more actionable (because it deals with specifics) as well as being more sensitive to the needs of the community.

**Figure 5:**
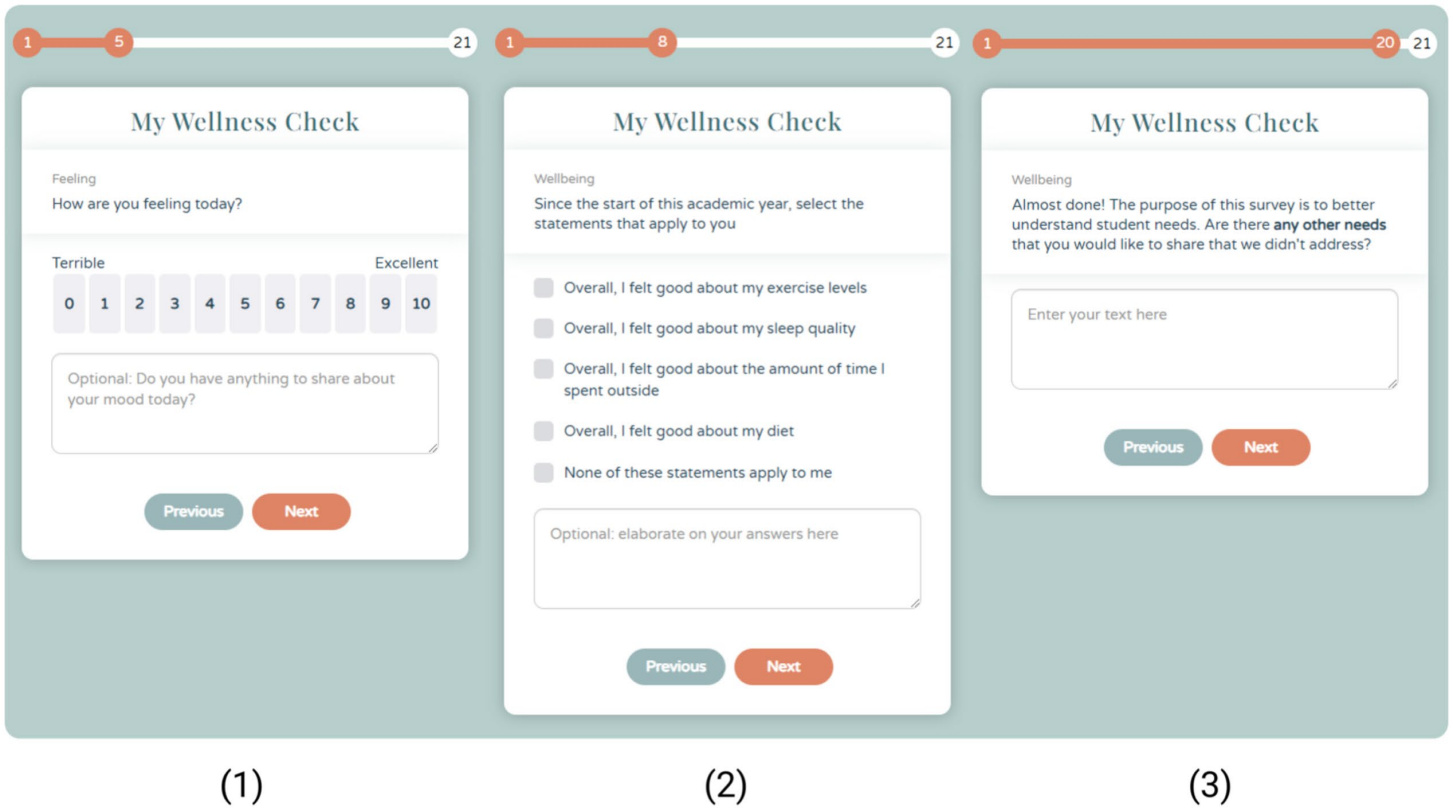
A selection of images depicting the appearance of the survey experience. The first screen shows a *rating* item about life satisfaction, the second, a *checkbox* item about physical wellbeing, and the third item shows a *free text* item about their additional wellbeing needs.

Therefore, we used factorized models of wellbeing as an organizing principle to help identify concrete and specific questions that could support community action. Figure 5 shows how various theoretical factors underpinning wellbeing may manifest within our context. For instance, many different models of human wellbeing recognize Material Wellbeing as an important factor of wellbeing (e.g., Sirgy, 2018). However, what material wellbeing means is likely to differ from one context to the next. In the context of wellbeing during COVID-19, for instance, we asked about the ergonomic quality of home workspaces—which can be seen as a causal indicator (Wong and Law, 1999)—as part of an effort to assess the influence of the home working environment on wellbeing. According to Mackenzie et al. (2011), it is specifically these sorts of causal indicators that belong of survey instruments.

Across all iterations, the My Wellness Check assessment considered a diverse range of indicators for community wellbeing: academic experience, anxiety, autonomy, behavior, belongingness, competence, coping strategies, COVID-19 measures, depression, drugs and alcohol, exercise, expected university support, finances, home working environment, life satisfaction, loneliness, mood, motivation, nutrition, optimism, overall physical health, personal growth, purposefulness, remote education, sleep, study performance and subjective mood. Additionally, various surveys were consulted in constructing the assessment items, including “The Warwick-Edinburgh Mental Wellbeing Scale (WEMWBS)” (Tennant et al., 2007), “PERMA-profiler” (Butler and Kern, 2016), “Satisfaction with life Scale (SWLS-5)” (Diener et al., 1985; Pavot and Diener, 2009), “Harmony in Life Scale (HILS-3)” (Kjell and Diener, 2020), “World Health Organization (WHO-5)” (Topp et al., 2015), “Psychological Wellbeing Scale (PWB)” (Ryff and Keyes, 1995; Ryff and Singer, 2006), “Wisconsin Longitudinal Study (WLS)” (Piliavin and Siegl, 2007), “Midlife in the United States (MIDUS)” (Radler and Ryff, 2010), “National Survey of Families and Households (NSFH II)” (Springer and Hauser, 2006), “College Student Subjective Wellbeing Questionnaire (CSSWQ)” (Renshaw and Bolognino, 2014), and “Student WPQ” (Williams et al., 2017).

#### Community-led design of survey

The design of our community wellbeing assessment combined traditional psychological methods for survey development (Boateng et al., 2018) with a variety of human-centered design approaches, particularly community-led design methods (Costanza-Chock, 2020). By community-led design, we specifically mean that we involved community members and community leaders in the informal design and informal evaluation of the assessment instrument (see Lomas and van der Maden, 2021). Rather than approaching this in a strictly systematic manner (typical of psychological survey development), we encouraged various levels of university leadership to “weigh in” on the types of questions to be asked (typically in response to a proposed concrete example). By engaging with community leaders, we were able to create a greater sense of investment and enthusiasm for the implementation of the assessment. Additionally, there were three concepts central to the development of the instrument: context-sensitivity, actionability, and assessment experience—see Box 4.

To balance out the needs of community leaders with the needs of the community at large, we also put significant effort into gather diverse perspectives across the many iterations of the survey. At first, we used informal, semi-structured interviews with about 15 students and 7 staff members to gather perspectives on current needs and ideas regarding the academic experience and overall community wellbeing. These interviews were focused on identifying concrete and specific indicators associated with different theoretical factors of wellbeing. Together with the priorities of university leadership, these community interviews helped inform the focus of our initial set of survey questions. Once an initial survey experience was developed, a sample of about 40 diverse students were asked to complete the entire survey over video chat. While offering informed consent and promising anonymity, we encouraged participants to comment aloud on individual survey items and give critical feedback. All content data was discarded for privacy reasons. However, this observational method was helpful for improving the relevance of the questions, reducing ambiguity about the meaning of questions and generally ensuring that important topics were not overlooked. Over the course of this development (which took approximately 4 weeks), many subtle iterations to the survey were made to ensure appropriate pacing and sequencing.

This iterative design and survey development continued even into the subsequent deployments of the survey, discussed in the following section. An example of the mobile user interface is presented in Figure 5; this shows the effort taken to create a motivating and positive survey experience. It also shows the tight integration of quantitative data collection procedures with opportunities to gather the voice of respondents in free text boxes.

After the initial deployments of the survey, statistical data about individual items made it possible to identify items that well predicted our central measure Life Satisfaction and items that did not. Furthermore, following the survey, all respondents were offered the opportunity to leave critical feedback about improving the survey. This strong focus on community-led iteration was hypothesized to create a better survey experience and to produce a more sensitive sensor of community wellbeing. As will be discussed, these hypotheses were tested through a controlled experiment.

### Deploying the assessment of community wellbeing

This section presents an overview of the deployment of our assessment of community wellbeing to 27,270 students and 6,347 staff members staff members (total university population during the last iteration, November 2021). Separate surveys were designed for students and staff. The number of participants and a summary of each iteration can be found in Table 1.

**Table 1:** An overview of the different iterations of wellbeing assessment conducted at Delft University of Technology.

Iteration	Date	*n*	Completion rate	# Q	# I
Staff 1	June 2020	2,776	85% (2328)	24	56
Student 1	June 2020	3,150	81% (2604)	25	79
Student 2	November 2020	3,409	80% (2841)	26	82
Staff 2	December 2020	1826	89% (1622)	22	76
Student 3	March 2021	2,877	77% (2221)	19	55
Staff 3	June 2021	2,376	84% (2006)	25	49
**Student 4**	**June 2021**	**2062**	**80% (1719)**	**19**	**79**
Student 5	November 2021	1835	81% (1492)	19	91

**Figure 6:**
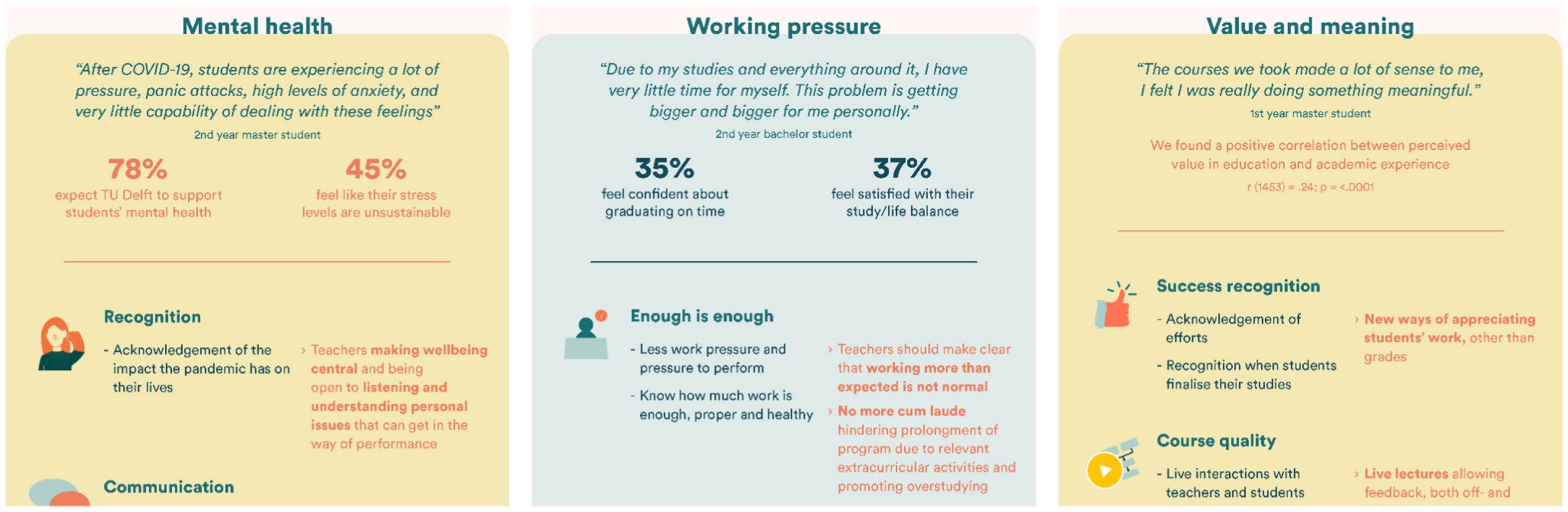
This figure shows parts of the infographic that was shared with all educators within the university, outlining both the quantitative but particularly the qualitative data. The infographic was shared in a university-wide newsletter and was accompanied by a short summary of the study and the message that this infographic could help inspire educators in creating an educational environment that supports wellbeing.

‘*# Q*’ refers to the number of questions included and ‘*# I*’ refers to the number of items included (some questions contained multiple items, like the checkbox item in Figure 6). The iteration involving the controlled experiment is highlighted in bold.

All students and staff received an email in both Dutch and English that invited them to participate in the study. The email contained a link that led them to an online version of the survey that could either be completed on a tablet, phone, or desktop. The welcome text of the assessment provided participants with information about the anonymity of their data (the limitations to guaranteeing their anonymity will be addressed in the discussion), the fact that the assessment was compliant with GDPR standards, and thus provided them with enough information to give their informed consent. All data were anonymized.

#### Quantitative results over time

For reasons of space, we focus on our longitudinal data on student results. Table 2 presents student wellbeing data over time, based on rating-type questions (i.e., requesting a rating on a scale from 0 to 10). Table 3 shares data listing the percentage of students selecting that they agree with a specific statement (checkbox items).

**Table 2:** An overview of data gathered in five iterations through scaled items about student wellbeing at Delft University of Technology.

		June 2020	October 2020	March 2021	June 2021	November 2021
	*n*	2,604	2,841	2,221	1719	1,492
Life Satisfaction	*M (SD)*	6.4 (1.9)	6.0 (1.9)	5.3 (2.0)	5.9 (2.1)	6.1 (2.0)
Mood	*M (SD)*	6.3 (1.9)	6.0 (1.7)	5.5 (1.9)	5.9 (2.0)	5.9 (1.9)
Physical Health	*M (SD)*	6.9 (1.8)	7.1 (1.6)	6.3 (1.9)	6.5 (1.9)	6.7 (1.9)
Studying At Home	*M (SD)*	5.5 (2.2)	5.7 (2.1)	5.3 (2.4)	5.4 (2.5)	-
Academic Experience	*M (SD)*	-	-	-	4.8 (2.2)	5.9 (1.9)
Studying on Campus	*M (SD)*	-	-	-	-	6.6 (2.1)

The range of each scale was from 0 (“Terrible”) to 10 (“Excellent”), except for life satisfaction which was from 0 (“Very dissatisfied”) to 10 (“Very satisfied”).

**Table 3:** Showing the proportion of students that agree with the statements presented as checkboxes.

		Percent saying yes				
		Jun. 2020	Oct. 2020	Mar. 2021	Jun. 2021	Nov. 2021
Belongingness	I feel part of a community at [university]	44	28	20	24	26
	I often feel lonely	31	40	42	36	36
	I feel like I belong at [university]	57	41	41	38	33
	It often feels like no one at [university] cares about me	21	21	25	24	24
	I often feel like I do not have anyone to talk to				18	28
	I feel that my fellow students care about me and each other				39	27
	I have a good bond with one or more of my fellow students				67	60
	I would feel comfortable letting a professor know if I need help				26	
	Often, I felt like I could be myself around my fellow students					47
	Often, I felt left out					12
Overall Wellbeing	Overall. I felt good about my exercise levels	45	44	34	44	45
	Overall. I felt good about my sleep quality	52	51	48	46	46
	Overall. I felt good about my diet	61	62	54	53	53
	Overall. I often felt down	46	46	59	44	44
	I often worry too much	58	65	58	58	63
	Overall. I felt good about the amount of time I spent outside			26	43	40
	I feel like my stress levels are unsustainable				39	45
	Often, I felt relaxed					19
	Often, I did not feel good about myself					32
Studies	I feel confident about graduating on time	50	45	42	42	35
	I am generally optimistic about the future	61	56	51	53	36
	I am happy with how I am performing in my studies	63	50	48	50	
	I am satisfied with my study/life balance	39	31	19	25	37
	I feel capable at what I do			35	39	
	I feel motivated to finish my current study program			57	58	58
	Overall, I felt I will be prepared to continue with my career successfully					34
	Overall, I felt satisfied with my online / offline balance					34

For example, the proportion of students that feel that they are a part of a community at Delft University of Technology has decreased by 20 percentile points within a year.

#### Qualitative results

In addition to statistical measures, we also designed the survey to support the collection of written text that represented the “voice” of students and staff. Open-ended questions were important for assessing community needs and also to gather possible actions that the university might take to help. These questions included: “What contributes to your sense of belonging and community at Delft University of Technology?;” “What aspects are you missing?” and; “Do you have ideas on how Delft University of Technology might help support student motivation?”

Open-ended questions were useful for eliciting concrete statements of student needs as well as serving as a source for specific ideas for the improvement of wellbeing. The quantitative data was useful for showing patterns across different university populations (e.g., could see the degree of physical health issues in international students v. local students). Written responses were often a source of specific ideas for organizational improvement. For instance: *“I really miss the in-between coffee chats with fellow students and the company. I want to see people”*; *“I really hope that hybrid learning can continue and that I can finish my master’s degree while being in my home country.”*; *“Organize silent discos with circles on the ground so that you can dance just in your own space!”; “I’m so happy to be back on campus, the facilities are awesome. However, it is hard to find a quiet place where I can talk in videocalls.”* and; *Because of* [COVID-19], *some know each other very well and others do not. It’s hard to join a group, especially if you do not know anyone at all. The university could help organize meeting groups for international students*.”

### Designing for community action

The previous section focused on the design of a sensor for community wellbeing. The purpose of this sensor was not just to generate a measure, but to serve in a cybernetic feedback loop that could motivate subsequent community actions—actions that could help contribute to improvements in community wellbeing.

Therefore, there were two core tasks required in designing for community action: 1) identify possible actions that could plausibly improve community wellbeing and; 2) motivate community actors to take appropriate actions. In practice, we made an effort to combine these together: when community actors were engaged in a process to help them identify useful actions for improving wellbeing, this was a key motivation for their subsequent actions.

Following each survey iteration, we held community-led design workshops with approximately 20 to 40 diverse online participants. Workshop participants included students elected to the student council, staff counselors (including psychologists and employees involved in mental health coaching), deans, upper administrators, the vice-rector, and various other students and staff all from across the university. Prior to each workshop, each participant was given several hundred written responses to review. With the instruction to identify unique needs and their ideas for how to help. At the workshop, small groups synthesized and discussed these lists of needs and ideas. Following a whole group discussion about the “doability” and “urgency” of different ideas, the lists of needs and ideas were compiled together for presentation to university upper administration. The aim was to analyze qualitative data to inform the communities about the wellbeing needs emerging from the survey and to collect ideas for improvement. To maximize the potential for action, we took special care to involve administrative decision-makers in reading and reviewing survey responses. Below, Table 4 highlights some of the ideas and the institutions that may be able to act in accordance with them.

**Table 4:** Many examples of ideas for action coupled with a potential actor (i.e., ‘a cybernetic processor’) and their respective areas of need as communicated back to the community.

Area	Idea for Action	Actor
Guidance	Organize collective day starts, cultivate a morning routine	University culture and sports center
	An effectiveness tracking tool to overview your work progress	Heads of Education and Student Affairs
	Group for simultaneously graduating students (SCRUM meetings)	Graduate mentors, teachers, program directors, Library, Heads of Education and Student Affairs,
	Motivate students to go outside (RSI prevention)	University culture and sports center, wellbeing taskforce
	More available counselors and psychologists	Career and Counseling Service, Heads of Education and Student Affairs, Student Council
	An online chat box for talking to student-psychologists	Communications dept., Career and Counseling Service, ICT
	Provide people buddy/study groups and guidelines on healthy routines	Academic counselors, Program directors, graduations progress at Education and Student Affairs
	A platform where students can share their tips and tricks for how to cope with the pandemic	Career and Counseling Service, Education and Student Affairs, Student Communications dept., Wellbeing taskforce
	Increase (online) contact hours with teachers and mentors	Program directors
Communication	Positive communication from departments, professors, teaching staff. Clear, regular, and motivational.	Communications dept., Student Communication dept., Department deans.
	COVID-19 website should be more up to date and accessible (including a weekly blog)	Communications dept., Wellbeing taskforce, and Career and Counseling Service
	Facilitate office hours and mentoring with digital tools like ‘Calendly’	Communications dept.,, Science center, X, Study and Student associations, and the Library
Workspace	More available, COVID-proof, working spaces on campus	Library, Heads of Education and Student Affair, Faculty deans, Facility Management, Faculty Secretary, Student Wellbeing Taskforce, and Alumni Office
	Support for home offices (with Wi-Fi etc.)	Process Manage, Faculties, Design Graduate projects
Social	Improved Peer Mentoring	Bachelor and Master coordinators and Communications dept.
	Online platform to meet other students (particularly for internationals)	Teachers and teaching assistants, Career and Counseling Service, Education and Student Affairs, Student Communications dept. and academic counselors
	Support for Community Organizers	Communications dept., Science Center, University culture and sports center, student and study associations and the library
	Online (drop-in) groups for activities (e.g., fitness at home)	University culture and sports center, Student Initiatives
	Online dinners and coffee moments	University culture and sports center and student and study associations
Health	45-min zoom meetings, not an hour	Teachers, schedulers, and teaching assistants
	Sport and culture courses online	University culture and sports center and the Executive Board
	Educational activities that can be done without a computer	Teachers and teaching assistants
Financial	Share models for how to deal with student loans in the future	Student deans and counselors
	Workshops on CV creation and jobfinding	Career and Counseling Service
	Promote jobs as student assistants while other opportunities are shutdown	Student council, Student Recruiting Services, Communications dept.
	Make financial advisors available/provide financial survival tips	Student dean

The list was constructed based on the data of the June and November 2020 surveys, at the height of the pandemic, meaning that students were not allowed to go to campus. The areas of need were: guidance, communication, workspace, social interaction, health, and financial. Note that this list is not exhaustive because in reality over hundreds of ideas have been cataloged and coupled to potential actors. Rather, this table is intended as a means to give the reader an approximation of the action space.

Motivating community action also occurred through the presentation of data to various stakeholders. For instance, following data collection events, data presentations were made to the executive board of the university and to the board of education. Several policies can be directly linked to the results of our analyses. For instance, the university organized a ‘Wellbeing Week’ with various activities related to the outcomes of the report (i.e., supporting sleep, exercise and socializing). More concretely, as we found that the home working environment was a strong predictor of wellbeing, the university funded a program to provide ergonomic chairs and desks. A subtler example came from the impact of many students expressing that they’d appreciate a more *human* communication approach—e.g., the dean sending out emails asking students how they were doing, in a very personal manner. This finding encouraged the university to provide guidance on altering the tone of voice in official emails. Next to administrative changes, action was also taken from a community perspective. For example, many PhD students that started in times of corona expressed they missed the opportunity to meet people and have “spontaneous social contact.” This inspired a program called ‘PhD Speed Dating’ where PhDs were assigned to a random person on zoom so they could chat and expand their social network.

Beyond these top-down policies, data were also used to motivate bottom-up community responses. Infographics were designed (see Figure 6) to communicate results to educators, staff and students at the university. These materials did not just incorporate quantitative survey data, but also the qualitative “voice” of students. Educators were invited to take these results in consideration when designing courses, lectures, and interactions with students. One responded: *“When something resonates with me and I empathize with it, I feel the urgency to act and implement improvements in my practice.”*

When measures against COVID-19 allowed it, community workshops were also organized in person. A community of researchers and designers were engaged in a workshop inspired by the World Café format, which is based on the belief that people within an organization, if put in a social environment open to dialogue and exchange, can find solutions even to complicated issues (Löhr et al., 2020).

**Figure 7:**
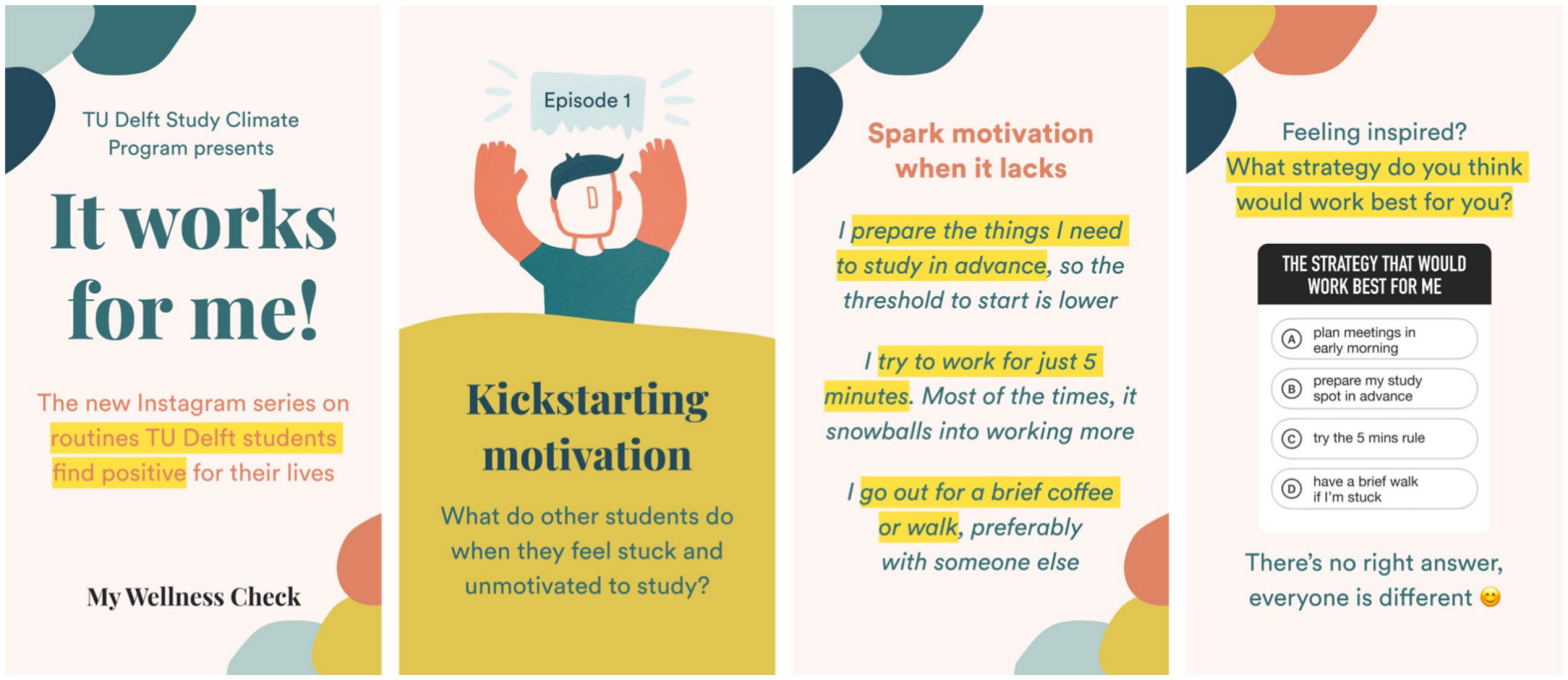
This figure shows a selection of the ‘It works for me!” wellbeing campaign launched in cooperation with local student associations. The goal was to gather routines or rituals from the results of the survey that students use to promote their wellbeing and share them with their peers.

We promoted and designed initiatives enabling the student community to take action and create impact on itself as well. All answers to the question “What daily routines are working well for you?” were collected and analyzed. This resulted in several visuals, which were shared in episodes once a week by study associations in their social media accounts, see Figure 7. The four episodes covered important student topics that emerged from the survey responses’ analysis itself and consisted of first-person sentences about positive routines. The goal was to inspire students with routines that worked for their peers, hence having a higher chance to work for them as well. Other bottom-up results include student projects focused on wellbeing. One student, for instance, created a recommendation system to help students optimize their living situation on a budget and promoted this to thousands of students.

### Experimental evaluation

A quantitative evaluation of our overall system remains challenging—for instance, it would be largely infeasible to conduct a controlled experiment involving multiple communities. For this reason, we have sought to quantitatively evaluate parts of the system. In this next section, we share the results of a controlled experiment conducted to compare our wellbeing assessment to other wellbeing assessments that were developed using standard psychological methods (Boateng et al., 2018). Because our community-led design methods so actively engaged diverse stakeholders in our community, there is a possibility that it may have led to reduced measurement efficacy. We chose the Warwick-Edinburgh Mental Wellbeing Scale (WEMWBS) and the College Student Subjective Wellbeing Questionnaire (CSSWQ) because they are widely used wellbeing assessment instruments and suitable in the context of our survey intervention. That is, we could not expect students to complete a full Positive and Negative Assessment Scale (PANAS)—to point to another widely used assessment instrument. It would have been too burdensome and distinct from the survey experience they were used to after four iterations (i.e., MWC).

In comparison to existing and validated assessments, we aimed to test the following two hypotheses. We predicted that our context-sensitive assessment of wellbeing would achieve:

1. improved prediction of life satisfaction (a core measure of human wellbeing)
2. improved measures of user experience

These hypotheses were tested by randomly assigning samples of the research population to answer one of three questionnaires (WEMWBS, CSSWQ, MWC) after which their evaluation of their experience had been compared. This controlled experiment was conducted during the fourth iteration of the student survey, June 2021, see Table 5.

**Table 5:** An overview of the different iterations of wellbeing assessment conducted at Delft University of Technology.

Iteration	Date	*n*	*Completion rate*	# Q	# I
Staff 1	June 2020	2,776	85% (2328)	24	56
Student 1	June 2020	3,150	81% (2604)	25	79
Student 2	November 2020	3,409	80% (2841)	26	82
Staff 2	December 2020	1,826	89% (1622)	22	76
Student 3	March 2021	2,877	77% (2221)	19	55
Staff 3	June 2021	2,376	84% (2006)	25	49
**Student 4**	**June 2021**	**2,062**	**80% (1719)**	**19**	**79**
Student 5	November 2021	1,835	81% (1492)	19	91

#Q refers to the number of questions included and #I refers to the number of items included. The iteration that is discussed in this methods section is highlighted (Student 4). We have also included the iteration that took place after that because it was considered relevant for our discussion, particularly with regards to the section about sharing data back to the community—regardless, there were no experimental conditions to that iteration.

### Procedure

2,062 student participants were randomly assigned to different versions of the questionnaire: 12.5 percent of all participants would receive the WEMWBS, 12.5 percent would receive CSSWQ and the remainder would receive My Wellness Check (75 percent). These proportions were chosen because we have conducted our study in a real-world setting, meaning that the objective of the survey had to remain true to the initial goal—gathering data on student and staff wellbeing during COVID-19 to inform institutional action. The WEMWBS and CSSWQ were chosen because they are frequently used and validated measures of global and domain-specific wellbeing.

Prior to beginning the experimental questions, all participants answered a common question about their life satisfaction. Following the experimental questions, participants were asked to complete seven questions about their questionnaire experience based on Stocké and Langfeldt (2004) and Baumgartner et al. (2021).

### Experimental results

Table 6 shows that our context-sensitive assessment improved the overall sensitivity of the assessment and enhanced the survey experience for participants. To calculate sensitivity, we used a regression model to predict individual Life Satisfaction scores using the responses to questions from the three surveys. My Wellness Check (MWC) produced a higher *R^2^* (a measure of predictive fit) than the WEMWBS or CSSWQ. Including all MWC items in the model produces an *R^2^* of 0.75 while restricting the model to only the checkbox items (not the 0–10 scale questions) still produced an *R^2^* of 0.53, exceeding the *R^2^* of a model with all items in the WEMWBS (*R^2^* = 0.51) and a model with all items in the CSSWQ (*R^2^* = 0.42). Then, to compare participant ratings of the survey experience, a MANOVA showed a significant positive difference (*p* < 0.0001) between My Wellness Check (MWC) and WEMWBS and CSSWQ across all items listed in Table 6. The sole exception was that MWC was significantly more exhausting (*p* < 0.0001), see Table 6. This shows that participants taking the MWC survey found the experience to be of significantly greater value, significantly more engaging, significantly more worthwhile and significantly more fun. All statistical tests were conducted using JMP 16.

**Table 6:** An overview of the experimental results during iteration four.

	MWC	WEMWBS	CSSWQ
Correlation with Life Satisfaction expressed by R2	0.75	0.51	0.42
How satisfied were you with this questionnaire?	6.9 (1.7)	6.2 (2.0)	5.9 (1.9)
This questionnaire was of high quality	3.8 (0.8)	3.3 (1.0)	3.1 (0.9)
Completing this questionnaire was of some value to me	3.3 (1.0)	2.9 (1.1)	2.6 (1.0)
Completing this questionnaire was engaging for me	3.2 (1.0)	2.8 (1.1)	2.7 (1.0)
Completing this questionnaire was exhausting	2.2 (1.0)	1.8 (0.9)	1.9 (1.0)
Completing this questionnaire was worthwhile	3.5 (0.9)	3.2 (1.0)	3.0 (0.9)
Completing this questionnaire was fun	2.9 (1.0)	2.7 (0.9)	2.6 (1.0)
Number of questions	17	16	14
Average completion time in minutes (SD)	7:51 (9:45)	5:47 (7:17)	5:22 (9:40)

This experiment was twofold. Firstly, the top row shows the correlation each model had with life satisfaction expressed by their effect size (R2)—i.e., the degree to which they were able to predict life satisfaction. Secondly, the table shows a comparison between the questionnaire experience of My Wellness Check (MWC), Warwick-Edinburgh Mental Wellbeing Scales (WEMWBS), and College Student Subjective Wellbeing Questionnaire (CSSWQ). The range for the question about satisfaction was 0 (“Very dissatisfied”) to 10 (“Very satisfied”). For the other questionnaire experience questions, the range was from 1 (“Totally disagree”) to 5 (“Totally agree”).

## Discussion

The aim of this article is to demonstrate an approach to designing systems for improving community wellbeing. Based on a proposed framework for designing AI systems, we highlight the value of cybernetic theory when designing intelligent systems that involve complex human communities—in the sense that AI theory helps us to understand that feedback loops are a key feature of complex systems, and that involving humans in the design of feedback loops is necessary to create intelligent systems.

Based on this theoretical background, we share a case study in which we design an intelligent feedback loop to promote university student and staff wellbeing during COVID-19. Our work focuses on the use of community-led and human-centered design activities to produce “sensors” of wellbeing (a context-sensitive wellbeing assessment), “actuators” of wellbeing (a space of action that can be taken by different stakeholders in our community), and “processors” of wellbeing (which enable the transformation of sensor data into action). In our case study, we describe the longitudinal fluctuation of community wellbeing over 2 years of the COVID-19 pandemic and explain the range of actions taken in response. To evaluate our efforts, we also share the results of a controlled experiment which indicate that our wellbeing assessment has improved sensitivity to wellbeing and provides an improved user experience in comparison to other “off-the-shelf” wellbeing assessment instruments. Our work demonstrates that community-led and human-centered design approaches may aid in the development of systems that can enhance and support wellbeing. Below, is a general schematic of our framework. Note that these steps apply to any complex system be they predominantly artificial or humane.

The remainder of our discussion shares a vision for describing how our “AI for Wellbeing” approach might generalize to other complex systems, including online social media systems and national governments. We then discuss several important limitations to this approach. Finally, we reflect on the relative merits of “cybernetic thinking” in the design of systems that seek to integrate human and machine intelligence.

### A generalized vision for designing intelligent systems to support community wellbeing

The case study in this article is specific to the context of our own university during the COVID-19 pandemic. The approaches and methods may be generally applicable to other universities or organizations that seek to prioritize community wellbeing. Beyond this, our framework and methods show promise for guiding the design of wellbeing feedback loops within other complex sociotechnical systems. In other words, our approach is not necessarily a blueprint for the next “COVID-22” but rather a way to understand how systems can deal with novel or urgent phenomena that affect the global society at large.

For instance, approaches taken here may offer insights for the integration of human wellbeing into the optimization of contemporary social media platforms like Facebook. To provide context, the CEO of Meta said: “we feel a responsibility to make sure our services aren’t just fun to use, but also good for people’s wellbeing” (2018). This statement introduced a new “wellbeing” metric called Meaningful Social Interactions (MSI). Three years later, however, the ‘Facebook Files’ (Zuckerberg, 2018; Hagey and Horwitz, 2021) showed that there are still many aspects of social media services that harm user wellbeing. Our work demonstrates a system design approach and community-led design methods for human wellbeing feedback loops that may be useful in the design of social media services and other sociotechnical systems. For instance, Facebook’s MSI metric could be refined and expanded with wellbeing data collected through the community-led design methods and system design approach described in this article.

Our work may also generalize to societal governance, in general. During the COVID-19 pandemic, wellbeing in Europe fell to its lowest level in 40 years (Allas et al., 2020). Wellbeing is often not explicitly valued in discussions of economic growth and decline. However, McKinsey and Co proposed a model of the monetary value of wellbeing by considering how much additional income a person would need to receive in order to raise their wellbeing by a desired amount. With this model, McKinsey estimated that wellbeing losses during the COVID-19 pandemic cost more than three times as much as the economic losses (i.e., reduction in GDP).

Increasingly, national governments are shifting from a single-minded focus on economic growth and turning to a more integrated ‘wellbeing economy’ focus (Fioramonti et al., 2022). Since the country of Bhutan changed its constitution in 2008 (Ura et al., 2012) to focus on “Gross National Happiness,” the idea of wellbeing-based governance has become an intense topic of research. The Organization for Economic Co-operation and Development (OECD) promotes and maintains a measure of country-wide happiness and wellbeing that is used for ranking and policy purposes (Mizobuchi, 2014). Clearly, there are moves to make citizen wellbeing a more explicit measure of government success.

Here, we wish to communicate a design vision for governance for wellbeing that is focused on the experience of citizens. What do we want it to *feel* like to have governments or even smaller organizations work to maximize the wellbeing of their people? After all, there are always risks that come from focusing too much on optimizing a single metric (Rambur et al., 2013; Stray, 2020; Thomas and Uminsky, 2020). We turn to metaphor to communicate our design vision (Hekkert and van Dijk, 2011). An AI-based optimization of human wellbeing may feel unnerving, as though we have put a machine in charge of running society. Instead, our vision for optimizing societal wellbeing aims to feel more like a deliberative democratic process. Perhaps governments could use systematic wellbeing assessments as a participatory ritual (akin to voting day) to make it easier to “listen to the voices of the people.” Then, we envision that the collective review of citizen needs and wants could feel more like deliberative, “town hall” democracy: a messy, time-consuming but intensely social process of figuring out *what do people need?* and *what actions can be taken to help?* Our case study shows the potential for using human-centered and community-led methods to optimize wellbeing in organizations large and small; the above design vision aims to communicate how “AI for Wellbeing” might be extended to “governance for wellbeing” in a humanistic manner.

### Limitations

The goal of an “AI for Wellbeing” system is to improve human wellbeing. Designing such a system requires, foremost, measurements of human wellbeing. But it also requires the ability to take actions in response to measurements. In an ideal world, the actions taken in response to wellbeing assessments would be (1) observable, (2) theoretically grounded (or to have a known mechanism of action and some predicted effect), and (3) empirically evaluated. However, in the case of a university during the COVID-19 pandemic, these criteria were not met. It was very difficult to know precisely what actions community members took in response to the assessment data. Further, few actions had a clearly defined theoretical model of how they were likely to impact wellbeing. Finally, none of the actions taken were evaluated statistically. Indeed, even if some actions were evaluated, there is little to suggest that they would have had the same effect at another point in time. As a result, it was difficult to evaluate the efficacy of our overall system. In other words, whether the human-centered activities conducted were the best *best of all possible* actions is indeterminable nor was it verifiable whether our approach was the *optimal* approach.

While we cannot make causal claims about the benefits of a wellbeing feedback loop, it may be possible to observe the functioning of our system like a prototypical cybernetic system, a thermostat. In a thermostat, a heater will stay on until the temperature reaches a desired range. In our case, once wellbeing returned to a range deemed “normal,” the system goal had been reached and the university was able to shift resources to “business as usual.” The community motivation for promoting wellbeing is analogous to the heater in this analogy. When wellbeing fell below a certain level, the university community was motivated to take a wide variety of actions. When wellbeing rose above an acceptable level, the motivation to focus on wellbeing was diminished. Like a thermostat, My Wellness Check turned up the motivation for action while the assessed need was high and reduced the motivation when the assessed need was low.

Designing this in a real-world university setting involved more than creating technologies, developing surveys, and executing human-centered design methods. It also required a messy, informal and unscientific political engagement by ourselves, as researchers and designers. This engagement was essential for getting buy-in and participation from multiple university stakeholders. Yet, through the dozens of meetings necessary to implement this system, we were able to leverage the community expertise of, for instance, psychological counselors, student advisors, human resource personnel, student council members and administrative leaders. This process is vastly more involved than simply “keeping humans in the loop” within a technical system. This limitation (or feature) will be relevant to the design of other intelligent systems for improving wellbeing within large-complex social environments: messy, democratic political processes may be required in addition to software development and user interface design. This creates new opportunities and demands for the appropriate role of human-centered designers in large, complex socio-technical systems.

### Design thinking, AI thinking, and cybernetic thinking

In this section, informed by our case study, we discuss our perspective on the design of AI systems applied within complex human systems. In the field of human-centered design, “design thinking” is a process for creative problem solving that is often used in the design industry (Pressman, 2019). In parallel, “AI thinking” can be described as a process for computational problem solving that is often used in the AI industry; in rough strokes, “if there is a problem, AI may be the solution.” However, many real-world problems are far more complex than AI algorithms can handle, particularly when AI is conceived as a fully autonomous agent. These real-world problems might include emotional engagement with other human participants or negotiating values or ethics. As a result, “AI thinking” has the potential to result in negative outcomes when it focuses AI designers on the production of fully autonomous systems that replace human intelligence with computational intelligence. A narrowed focus on algorithmic competence can result in the design of disembodied AI systems that fail to respect or leverage existing human capabilities in real-world systems (Gillespie, 2014; Krippendorff, 2021; Pangaro, 2021). For instance, “AI thinking” has produced product offerings promising to use complex data to provide medical diagnoses or educational recommendations. These offerings often fail because human doctors understandably distrust a “black box” diagnosis, just as teachers tend to distrust a “black box” curriculum selection (London, 2019; Wang et al., 2020). Instead, systems work better when they are not designed to be fully autonomous, but rather designed to provide services that can couple with existing workflows in an “unremarkable” manner (Yang et al., 2019).

“Human-centered AI” offers the opportunity to design AI systems designed to work in concert with humans, not just to replace them. Humans will remain better at understanding people and responding to their emotional needs for the foreseeable future. Complex ethical or value-laden decisions will continue to require human stakeholders to negotiate. AI design methods need to consider the limitations of AI systems and design AI systems capable of working in concert with humans and existing organizations—supporting humans in their decision-making, rather than seeking to replace them. If AI systems are to be used by humans, AI systems need to be designed to meet the needs of human users.

However, we suggest that there are important conceptual issues that emerge from trying to strictly distinguish between artificial intelligence and human intelligence. Many artificial processes, rules or algorithms exist within organizations that are designed to produce intelligent outcomes. Should the artificial design of intelligent human processes be considered artificial intelligence? If artificial intelligence is defined as “the artificial design of intelligent processes,” then this means that artificial intelligence does not require computational algorithms. Instead, algorithms might be merely written down and executed by humans. For example, “mastery learning” is an educational method involving a simple algorithm: if students demonstrate mastery on a topic test, they can proceed to the next topic, otherwise they are to continue to learn and master the topic at hand (Bloom, 1973). Mastery learning can be supported by computers, but it can also be implemented as a non-computational cybernetic feedback loop (e.g., just with teachers and paper tests).

Artificial intelligence could be defined as any kind of intelligent information process that is artificially designed—whether the process uses silicon microprocessors. This would broaden the scope of artificial intelligence to include all kinds of governing systems, not just those that rely on advanced computers. Consider the example of an autopilot; in the context of a self-driving car or even in a modern airplane, autopilot is certainly classified as a type of artificial intelligence. However, the first autopilot for an airplane was a mechanical system and was invented in 1912. If we take AI to mean “intelligent process that is artificially designed,” then the implication is that there is a great deal of AI that does not involve computers. This could have far-reaching implications for how we think about AI systems and their impact on society.

For the sake of convention, some may wish to adhere to a popular conception of artificial intelligence that might be described as “an autonomous algorithmic system that uses advanced computational techniques to accomplish non-trivial goals in a manner that precludes human intelligence.” In this case, the artificial design of intelligent processes that do not meet this definition might be termed “intelligent system design,” rather than “artificial intelligence.” Importantly, even non-computationally focused work may still contribute to the *field* of artificial intelligence, particularly when it demonstrates the application of artificial intelligence theory and methods to the design of intelligent systems.

In comparison to Design Thinking or AI Thinking, “Cybernetic Thinking” describes the design of intelligent systems, where the intelligence in the system relies on sensor/actuator feedback loops (van der Maden et al., 2022). By focusing on the design of information feedback loops, where a system’s performance is used to modify the system’s behavior, Cybernetic Thinking can be applied to the design of any goal-driven system, whether it is computational or not. For example, cybernetic thinking might be used in the design of educational systems (as in the description of mastery learning, above) or in employee performance reviews, where indicators of employee performance are used to modify ongoing performance. Cybernetic thinking may be valuable because it focuses on the dynamics of whole systems (including humans and machines), rather than naively focusing on popular computational algorithms. In our case, we found cybernetic thinking to be invaluable in the design of feedback loops to promote community wellbeing.

## Conclusion

Wellbeing is not just an individual concern, but a community and a societal concern. By designing a system to assess and support community wellbeing in the times of COVID-19, we have demonstrated how to systematically prioritize wellbeing as an explicit objective within large, complex social systems. Our work makes the following key contributions:

First, based on theories of artificial intelligence and cybernetics, we contribute an approach to designing feedback loops to support human wellbeing at a community scale. This approach is highly relevant to sociotechnical systems that have large numbers of individuals. In the context of our case study, we are working in a very large university with over 30,000 students and staff.

Second, we contribute a specific case study applied to the context of COVID-19. This case study provides practical examples of the application of our approach, such as the community-led design of online surveys to generate valuable feedback in the form of wellbeing data from our university community. This feedback is then fed back to the community in the form of qualitative assessments of needs and a summary statistics providing visual representations of how wellbeing changed over time and across sub-communities (e.g., academic and non-academic staff).

Third, we contribute an approach for using human wellbeing data to inform sociotechnical system design. We use our wellbeing data to generate insights and recommendations for improvements to our university’s COVID-19 response. For example, we provide action recommendations to particular stakeholders in the university. This is an important contribution as it provides a concrete example of how wellbeing data can be used to improve sociotechnical systems.

Finally, the result of adopting a cybernetic framework and using human-centered and community-led design methods, is the development of a novel *context-sensitiv*e wellbeing assessment. To evaluate our instrument, we conducted a controlled experiment: in comparison to other validated wellbeing assessment instruments, we found that our context-sensitive wellbeing assessment was more highly rated by participants and also demonstrated stronger predictive validity. We also present qualitative evidence showing that our assessment yields more “actionable” data for motivating institutional and community action.

In our approach to designing interactive systems to support wellbeing, we have shifted from a focus on individual user needs to designing for communities and institutions. We have also shifted our thinking from designing a static product to designing an intelligent product-service system—a system designed to operate as a cybernetic loop within a large and complex socio-technical system. Finally, our mindset shifted as we accepted that we were not the experts leading the design so much as facilitators of a community-led design process. These shifts may be subtle, but they represented an enormous leap from our initial perspectives on applying HCI, design and AI methods to create tools to support wellbeing during COVID-19. Our argument is that any future work on aligning AI systems with values like wellbeing and democracy will benefit from a similar process as the one presented in this paper.

## Data availability statement

The original contributions presented in the study are included in the article/supplementary material, further inquiries can be directed to the corresponding author.

## Ethics statement

The studies involving human participants were reviewed and approved by HREC TU Delft. The patients/participants provided their written informed consent to participate in this study.

## Author contributions

The research is primarily conducted by WM and DL who also contributed most of the writing with guidance and insight from PH. All authors contributed to the article and approved the submitted version.

## Conflict of interest

The authors declare that the research was conducted in the absence of any commercial or financial relationships that could be construed as a potential conflict of interest.

## Publisher’s note

All claims expressed in this article are solely those of the authors and do not necessarily represent those of their affiliated organizations, or those of the publisher, the editors and the reviewers. Any product that may be evaluated in this article, or claim that may be made by its manufacturer, is not guaranteed or endorsed by the publisher.
